# Life in the fast lane

**DOI:** 10.7554/eLife.35029

**Published:** 2018-02-20

**Authors:** C Loren Buck

**Affiliations:** 1 Center for Bioengineering InnovationNorthern Arizona UniversityFlagstaffUnited States; 2 Department of Biological SciencesNorthern Arizona UniversityFlagstaffUnited States

**Keywords:** garden dormice, juveniles, hibernation, torpor, fat reserves, life history, Other

## Abstract

Dormice born late in the year start to prepare for winter sooner than mice born earlier in the year.

**Related research article** Mahlert B, Gerritsmann H, Stalder G, Ruf T, Zahariev A, Blanc S. 2018. Implications of being born late in the active season for growth, fattening, torpor use, winter survival and fecundity. *eLife* **7**:e31225. doi: 10.7554/eLife.31225

Life requires a lot of energy, and since food resources fluctuate throughout the year, animals need to allocate their energy wisely. Vital processes such as reproduction and self-maintenance often compete for limited energy supplies, and many animals face trade-offs, especially in colder climates (Lindström et al., 2005).

Mammals have evolved different strategies to cope with these challenges. Some hoard food or migrate to more favorable environments, while others can slow down their metabolism and reduce their temperature until they have reached a state of torpor or even hibernation. Torpor only lasts for part of the day or night, but hibernation can last for months (Ruf and Geiser, 2015). Both methods conserve energy – but they come with a caveat (Lyman et al., 1982).

Since most species stop eating and drinking during hibernation, they need to accumulate enough energy and fat reserves beforehand. Adult animals typically achieve this through intensive foraging or moderating their metabolic rate and body temperature (Sheriff et al., 2013; Concannon et al., 2001). However, comparatively little is known about how young animals prepare for winter. Animals often have several litters per year. For example, dormice produce two litters – one born early and one born late in the season – which means that the young from the second litter have much less time to fatten up and grow (Giroud et al., 2014).

If growth happens at the expense of fattening, the animals may not survive the winter should they exhaust their fat reserves. But, choosing fattening over growth could reduce their future reproductive success, since a larger body size is linked to a higher fertility (Dmitriew, 2011). Now, in eLife, Sylvain Giroud of the University of Veterinary Medicine in Vienna and co-workers in Vienna and Strasbourg – including Britta Mahlert as first author – report how the second litter of garden dormice make up for their late arrival (Mahlert et al., 2018).

To assess how the two litters economize and allocate their energy supplies, Malhert et al. followed them as they grew up, and entered and reemerged from their first hibernation. Throughout that period, the researchers monitored and compared their body temperature, food intake (both litters received the same amount of food) and energy use, and also their body size and body mass (which served as a proxy for fat content; Dark, 2005).

Results showed that all animals invested in growth first before they started fattening. However, the second litter developed twice as fast as the first one, and also accumulated fat at a faster rate, albeit at the expense of retaining lower fat reserves. This indicates that irrespective of the timing of birth, the dormice invest in both growth and fattening in a stepwise fashion, but prioritize growth.

So, how do late-born dormice speed up their growth and fattening, and what are the hidden costs of doing so? To investigate this further, Mahlert et al. evaluated the pre-hibernation patterns of food consumption and torpor. All of the animals consumed more food prior to hibernation, but the second litter already started to increase the food intake two weeks after weaning, with a peak at four weeks. The first litter, however, only started to eat more from six weeks onwards, and reached their maximum at 10 weeks.

As winter approached, both litters fell into a daily torpor to conserve energy, but the late-born animals used it more frequently. Torpor was rarer during growth, but increased during fattening and peaked when both phases were complete. This suggests that torpor is incompatible with growth, which has also been observed in the Siberian hamster (Scherbarth et al., 2015). Moreover, the reduced body temperature and metabolic rate during torpor may help to gain and conserve fat during pre-hibernation (Sheriff et al., 2012; Sheriff et al., 2013).

When Mahlert et al. restricted access to food prior to hibernation, both sets of litters used torpor more frequently, and ultimately grew and fattened at the same rate and to the same degree as the animals that had free access to food. Collectively, these results suggest that the consequences of being born late are negligible, and that the second litter uses both energy intake and conservation strategies to catch-up with their early-born peers.

It is less clear why early-born dormice do not maximize their rate of growth and fattening, but it could be that accelerated growth minimizes their survival or reproductive success, and should therefore be avoided whenever possible (Dmitriew, 2011). However, Mahlert et al. found that late-born females (even the food-restricted ones) were more successful at reproducing than early-born females. This suggests that animals born later in the season could have a faster life history and therefore grow, mature and reproduce more rapidly.

At first glance, it may seem that life in the fast lane does not have any disadvantages. However, we know that there is no such thing as a free lunch in biology, and animals that grow more quickly may die at a younger age. An accelerated growth rate could also reduce the overall reproductive success or perhaps affect the health and fertility of their offspring. We will need long-term studies, spanning several generations, to uncover any potential hidden costs of being born late and adopting a fast pace of life. A deeper insight into how trade-offs furthering growth affect an organism, will help us to better understand how the diverse life-histories of animals have evolved.
